# The complete mitochondrial genome of *Silurus asotus* (Siluriformes: Siluridae: *Silurus*) and its phylogenetic analysis

**DOI:** 10.1080/23802359.2019.1630335

**Published:** 2019-07-12

**Authors:** Na Yang, Yingwen Li, Zhihao Liu, Qiliang Chen, Yanjun Shen

**Affiliations:** Chongqing Key Laboratory of Animal Biology, School of Life Sciences, Chongqing Normal University, Chongqing, China

**Keywords:** *Silurus asotus*, Siluridae, mitochondrial genome, phylogenetic

## Abstract

*Silurus asotus* is a widely distributed species, and mainly lives in the middle and lower layers of rivers, lakes, and reservoirs. In this study, we report the complete mitochondrial genome of *S. asotus*, which was 17,385 bp in length, containing 13 protein-coding genes, 2 rRNA genes, 22 tRNA genes, and a control region. Phylogenetic analyses indicated that *S. asotus* has a close relationship with *S. soldatovi*. This research work will provide some useful information on further molecular evolution studies of Siluridae species.

*Silurus asotus* is a worldwide distributed fish species. It usually inhabits in the bottom of the water, which grass clumps and water flowing slowly. *Silurus asotus* is a carnivorous benthic fish that feeds on small fish, shellfish, and frogs. Although, already many studies about Siluridae fishes are available (Wang et al. 2015; Xia et al. 2018), it is necessary to make more studies of this important economic species. Here, we report the complete mitogenome of *S. asotus* and its phylogenetic relationship.

The specimens of *S. asotus* were collected in Mudong of Chongqing (106°50′E, 29°34′N) and preserved in 100% ethanol in the museum of Chongqing Normal University until DNA extraction. The complete mitogenome sequence was 17,385 bp (GenBank: MK895951) and consist of 13 protein-coding genes, 2 rRNA genes, 22 tRNA genes, and a control region. Most genes were encoded on the H-strand, except ND6 and seven tRNA genes. The overall base composition of the mitogenome was 31.3% A, 26.2% T, 27% C, and 15.5% G. The total length of 13 PCGs was 11,408 bp. Similar to other vertebrates, all of the 13 PCGs in the mitogenome of *S. asotus* began with ATG, except COI gene with GTG. Incomplete stop codon is usual in vertebrate mitogenome and could be completed by posttranscriptional polyadenylation (Liu et al. 2013; Bai et al. 2018). There are seven genes (ND2, COII, COIII, ATP6, ND3, ND4, and Cyt b) that used two incomplete codons, T–– and TA–. Other five genes (ND1, COI, ATP8, ND4L, and ND5) used complete stop codon TAA, whereas ND6 used TAG.

To verify the phylogenetic position of *S. asotus* in family Siluridae, we utilized other nine Siluridae fishes and two outgroup species (*Loricaria cataphracta, Plotosus japonicus*). Maximum likelihood (ML) phylogenetic analysis was performed using MEGA 7 (Kumar et al. 2016) based on the concatenated 13 PCGs of these all species. Topological structure of the tree showed that *S. asotus* was clustered with *S. soldatovi* (BP = 100) (Figure 1). It is obvious that the phylogenetic position of *S. asotus* was closer to *S. soldatovi*.

**Figure 1.**
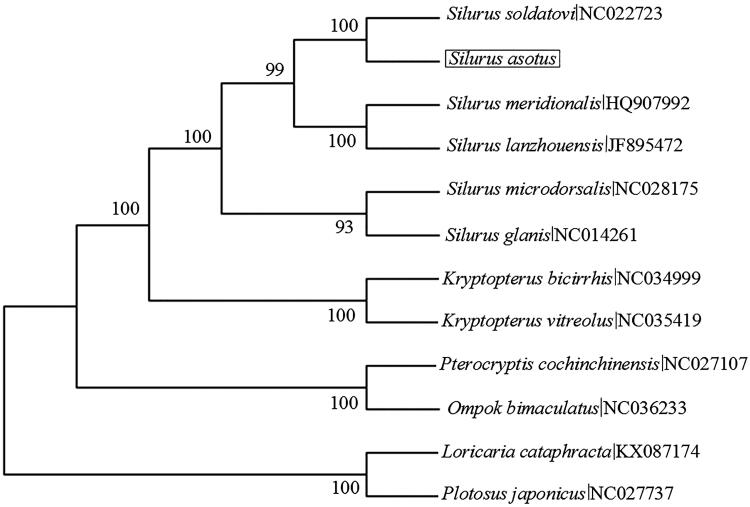
Maximum likelihood (ML) tree showing the phylogenetic position of *Silurus asotus* among Siluridae species based on a dataset of 13 PCG sequences. Only bootstrap support (BP) greater than 50% are shown above the lines.
